# Update on Vaccine-Derived Poliovirus Outbreaks — Worldwide, January 2020–June 2021

**DOI:** 10.15585/mmwr.mm7049a1

**Published:** 2021-12-10

**Authors:** Mary M. Alleman, Jaume Jorba, Elizabeth Henderson, Ousmane M. Diop, Shahzad Shaukat, Mohamed A. Traoré, Eric Wiesen, Steven G.F. Wassilak, Cara C. Burns

**Affiliations:** ^1^Global Immunization Division, Center for Global Health, CDC; ^2^Division of Viral Diseases, National Center for Immunization and Respiratory Diseases, CDC; ^3^Polio Eradication Department, World Health Organization, Geneva, Switzerland.

As of May 1, 2016, use of oral poliovirus vaccine (OPV) type 2 for routine and supplementary immunization activities ceased after a synchronized global switch from trivalent OPV (tOPV; containing Sabin strain types 1, 2, and 3) to bivalent OPV (bOPV; containing Sabin strain types 1 and 3) subsequent to the certified eradication of wild type poliovirus (WPV) type 2 in 2015 (*1*–*3*). Circulating vaccine-derived poliovirus (cVDPV) outbreaks* occur when transmission of Sabin strain poliovirus is prolonged in underimmunized populations, allowing viral genetic reversion to neurovirulence, resulting in cases of paralytic polio (*1*–*3*). Since the switch, monovalent OPV type 2 (mOPV2, containing Sabin strain type 2) has been used for response to cVDPV type 2 (cVDPV2) outbreaks; tOPV is used if cVDPV2 co-circulates with WPV type 1, and bOPV is used for cVDPV type 1 (cVDPV1) or type 3 (cVDPV3) outbreaks (*1*–*4*). In November 2020, the World Health Organization (WHO) Emergency Use Listing procedure authorized limited use of type 2 novel OPV (nOPV2), a vaccine modified to be more genetically stable than the Sabin strain, for cVDPV2 outbreak response (*3*,*5*). In October 2021, the Strategic Advisory Group of Experts on Immunization (WHO’s principal advisory group) permitted wider use of nOPV2; however, current nOPV2 supply is limited (*6*). This report updates that of July 2019–February 2020 to describe global cVDPV outbreaks during January 2020–June 2021 (as of November 9, 2021)^†^ (*3*). During this period, there were 44 cVDPV outbreaks of the three serotypes affecting 37 countries. The number of cVDPV2 cases increased from 366 in 2019 to 1,078 in 2020 (*7*). A goal of the Global Polio Eradication Initiative’s (GPEI) 2022–2026 Strategic Plan is to better address the challenges to early CVDPV2 outbreak detection and initiate prompt and high coverage outbreak responses with available type 2 OPV to interrupt transmission by the end of 2023 (*8*).

## Detection of cVDPV1

**FIGURE 1:**
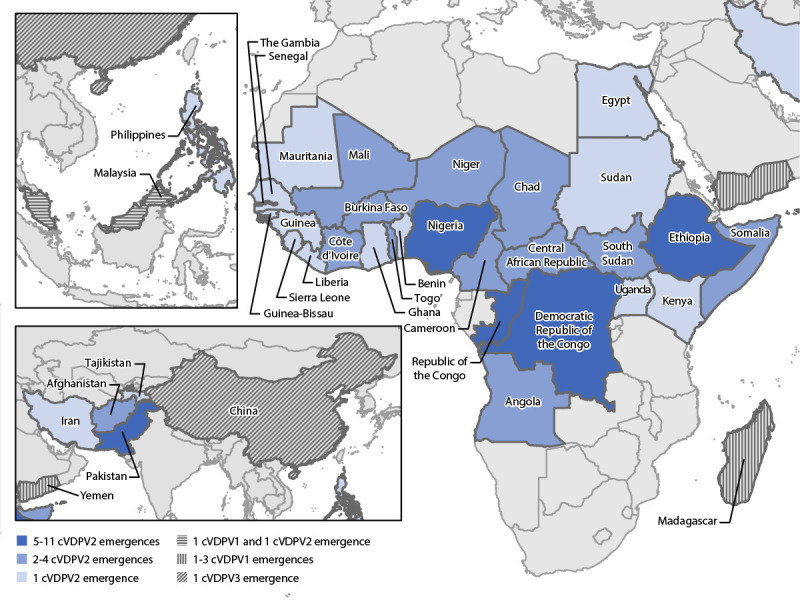
Ongoing circulating vaccine-derived poliovirus outbreaks — worldwide, January 2020–June 2021* **Abbreviations: **cVDPV = circulating vaccine-derived poliovirus; cVDPV1 = cVDPV type 1; cVDPV2 = cVDPV type 2; cVDPV3 = cVDPV type 3. * Data as of November 9, 2021.

The most recently detected poliovirus genetically linked to the cVDPV1 emergence (PHL-NCR-2)^§^ circulating during the previous reporting period was found in environmental surveillance samples (sewage) in Malaysia during March 2020 (*3*) (Table) (Figure 1). During this reporting period, three new cVDPV1 emergences were detected in Madagascar (MAD-ANO-1, MAD-SUE-1, and MAD-SUO-1). The YEM-SAD-1 emergence was first isolated from specimens collected during July 2019 from contacts of an acute flaccid paralysis (AFP) patient in Yemen; circulation was confirmed after the previous global update (*3*).

**TABLE:** Circulating vaccine-derived polioviruses detected, by serotype, source, and other selected characteristics — worldwide, January 2020–June 2021

Country	Outbreak/ Emergence designation*	Years detected^†^	Serotype	No. of detections^§^ January 2020–June 2021			Capsid protein VP1 divergence from Sabin OPV strain**(%)	Date of latest outbreak case, healthy child specimen, or environmental sample^††^
				From AFP cases	From other human sources (non-AFP)^¶^	From environmental surveillance		
Afghanistan	PAK-GB-1	2020–2021	2	225	36	271	0.7–3.4	Jun 9, 2021
	AFG-NGR-1	2020–2021	2	127	18	154	0.7–2.2	Jun 23, 2021
	AFG-HLD-1	2020–2021	2	4	0	5	0.9–1.7	Jan 28, 2021
Angola	ANG-HUI-1	2019–2020	2	2	0	0	1.3–1.5	Feb 9, 2020
	ANG-LUA-1	2019–2020	2	1	0	0	1.5	Feb 9, 2020
Benin	NIE-JIS-1	2019–2021	2	6	2	10	2.4–5.1	May 25, 2021
Burkina Faso	NIE-JIS-1	2019–2021	2	61	13	0	3.1–5.5	Jun 9, 2021
	TOG-SAV-1	2020	2	6	0	0	1.8–2.6	Oct 13, 2020
Cameroon	CHA-NDJ-1	2019–2020	2	3	0	0	1.4–1.9	Sep 20, 2020
	CAR-BER-1	2020	2	1	0	7	1.4–2.3	Sep 29, 2020
	CAR-BNG-1	2020	2	3	4	3	1.7–2.8	Jun 2, 2020
Central African Republic	CHA-NDJ-1	2020	2	3	1	0	1.4–1.7	Nov 4, 2020
	CAR-BER-1	2019–2020	2	1	0	0	1.3	Feb 5, 2020
	CAR-BNG-1	2019–2020	2	0	0	3	1.5–1.8	Feb 5, 2020
Chad	NIE-JIS-1	2019–2020	2	8	3	1	3.1–4.5	Aug 10, 2020
	CHA-NDJ-1	2019–2020	2	91	16	2	0.8–2.6	Dec 15, 2020
	CAR-BIM-3	2020	2	1	0	0	1.4	Oct 18, 2020
China	CHN-SHA-1	2020–2021	3	0	1	1	1.8–2.0	Jan 25, 2021
Côte d’Ivoire	NIE-JIS-1	2019–2020	2	63	27	175	2.9–5.1	Dec 23, 2020
	TOG-SAV-1	2020	2	1	0	0	2.0	Feb 10, 2020
Democratic Republic of the Congo	DRC-KAS-3	2019–2021	2	82	82	2	1.7–3.1	Apr 30, 2021
	DRC-MAN-2	2021	2	1	0	0	0.8	Jun 27, 2021
	DRC-TPA-2	2020	2	0	6	0	0.7–0.8	May 14, 2020
	DRC-EQT-1	2020	2	1	8	0	0.7–1.5	Sep 11, 2020
	CAR-BNG-1	2020	2	0	2	0	2.3	Oct 27, 2020
	ANG-LNO-2	2020	2	1	0	0	2.1	Feb 19, 2020
	ANG-LUA-1	2019–2020	2	2	0	0	1.0–1.3	Jan 29, 2020
Egypt	CHA-NDJ-1	2020–2021	2	0	0	11	2.1–2.5	Jun 8, 2021
Ethiopia	ETH-ORO-1	2019–2021	2	22	6	4	1.4–4.3	Mar 27, 2021
	ETH-ORO-2	2019–2020	2	2	0	0	1.3–1.5	Feb 18, 2020
	ETH-ORO-3	2019–2020	2	1	2	0	2.0–2.8	Oct 11, 2020
	ETH-ORO-4	2019–2020	2	1	0	0	2.9	Feb 23, 2020
	ETH-SOU-1	2020–2021	2	9	0	0	1.1–2.4	Apr 13, 2021
	ETH-SOU-2	2019–2021	2	5	0	0	2.1–3.0	Jun 24, 2021
	SOM-AWL-1	2020	2	2	0	0	1.5–2.3	Dec 14, 2020
	CHA-NDJ-1	2020	2	0	0	1	1.4	Dec 28, 2020
Ghana	NIE-JIS-1	2019–2020	2	11	10	34	2.9–4.1	Jun 16, 2020
Guinea	NIE-JIS-1	2020–2021	2	48	1	1	3.0–4.8	Apr 1, 2021
Guinea-Bissau	NIE-JIS-1	2021	2	2	0	0	4.1–4.5	Jun 27, 2021
Iran	PAK-GB-1	2020–2021	2	0	0	11	1.5–3.6	Feb 20, 2021
Kenya	SOM-BAN-1	2018, 2020–2021	2	0	3	2	7.2–7.6	Jan 25, 2021
Liberia	NIE-JIS-1	2020–2021	2	3	6	47	3.0–6.1	May 28, 2021
Madagascar	MAD-SUE-1	2020–2021	1	6	9	18	3.0–3.6	Jun 29, 2021
	MAD-SUO-1	2021	1	1	3	0	1.6–2.0	Feb 24, 2021
	MAD-ANO-1	2021	1	0	0	5	1.3–1.6	May 17, 2021
Malaysia	PHL-NCR-1	2019–2020	2	0	0	3	7.5	Feb 4, 2020
	PHL-NCR-2	2019–2020	1	3	0	10	3.4–4.0	Mar 13, 2020
Mali	NIE-SOS-7	2020	2	3	1	0	1.5–2.2	Jul 5, 2020
	NIE-JIS-1	2020	2	47	2	10	3.1–4.6	Dec 23, 2020
Mauritania	NIE-JIS-1	2021	2	0	0	2	3.9–4.0	Jun 30, 2021
Niger	NIE-JIS-1	2018–2020	2	11	2	11	2.8–5.1	Dec 8, 2020
	NIE-ZAS-1	2021	2	1	0	0	2.2	Jun 20, 2021
Nigeria	NIE-JIS-1	2018–2021	2	15	3	19	2.8–4.6	Jun 29, 2021
	NIE-SOS-8	2020	2	2	7	0	1.1–1.8	Sep 17, 2020
	NIE-ZAS-1	2020–2021	2	69	13	83	1.8–3.5	Jun 30, 2021
	NIE-SOS-7	2019, 2021	2	10	4	3	2.4–3.1	Jun 30, 2021
	NIE-KGS-1	2019–2020	2	1	0	1	1.4–1.5	Jan 26, 2020
Pakistan	PAK-GB-1	2019–2021	2	114	6	257	0.7–3.1	Apr 28, 2021
	PAK-TOR-1	2019–2020	2	0	1	1	1.1–1.5	Mar 4, 2020
	PAK-KHI-2	2020	2	0	0	4	0.7–1.0	Oct 14, 2020
	PAK-FSD-1	2020	2	10	1	8	0.7–1.2	Oct 13, 2020
	PAK-FSD-2	2020	2	2	0	0	0.8–1.4	Sep 29, 2020
	PAK-ZHB-1	2020	2	0	0	5	0.7–1.1	Oct 16, 2020
	AFG-NGR-1	2020–2021	2	12	2	59	0.7–2.3	May 18, 2021
	AFG-HLD-1	2020	2	2	0	0	1.3–1.4	Aug 24, 2020
	PAK-LKW-1	2020–2021	2	3	0	1	0.7–1.0	Jan 11, 2021
	PAK-KAM-1	2020–2021	2	0	0	4	0.7–0.9	Feb 9, 2021
	PAK-PWR-1	2021	2	0	0	2	0.8	Jun 14, 2021
Philippines	PHL-NCR-1	2019–2020	2	1	0	4	7.1–7.6	Jan 24, 2020
Republic of the Congo	ANG-HUI-1	2020	2	2	1	0	2.0–2.5	Nov 14, 2020
	DRC-KAS-1	2021	2	1	0	0	2.2	Jan 31, 2021
	CAR-BNG-1	2020–2021	2	0	0	4	2.3–2.6	Apr 14, 2021
	CAR-BER-1	2021	2	0	0	1	3.3	Jun 1, 2021
	ANG-LUA-1	2020	2	0	1	0	2.1	Oct 12, 2020
Senegal	NIE-JIS-1	2020–2021	2	14	30	13	3.8–5.7	Jun 14, 2021
Sierra Leone	NIE-JIS-1	2020–2021	2	15	16	10	3.4–4.6	Jun 29, 2021
Somalia	SOM-BAN-1	2017–2021	2	14	9	37	5.5–8.3	May 23, 2021
	SOM-AWL-1	2020	2	1	0	0	2.3	Aug 1, 2020
	ETH-ORO-3	2020	2	0	5	0	2.8	Sep 22, 2020
South Sudan	CHA-NDJ-1	2020–2021	2	56	24	11	1.3–3.0	Apr 8, 2021
	ETH-SOU-1	2021	2	1	0	0	2.2	Jan 8, 2021
Sudan	CHA-NDJ-1	2020	2	51	16	15	1.1–2.8	Dec 18, 2020
Tajikistan	PAK-GB-1	2020–2021	2	26	11	51	2.2–3.8	Jun 26, 2021
The Gambia	NIE-JIS-1	2021	2	0	0	14	4.0–4.6	Jun 24, 2021
Togo	NIE-JIS-1	2019–2020	2	6	8	0	2.8–4.1	July 9, 2020
	TOG-SAV-1	2019–2020	2	3	1	0	1.5–2.1	May 3, 2020
Uganda	CHA-NDJ-1	2021	2	0	0	1	4.0	Jun 1, 2021
Yemen	YEM-SAD-1	2019–2021	1	32	0	0	1.9–3.3	Jan 13, 2021
**Total cVDPV**	**—^§§^**	**—^§§^**	**—^§§^**	**1,335**	**423**	**1,412**	**—^§§^**	**—^§§^**

**Abbreviations**: AFP = acute flaccid paralysis; cVDPV = circulating vaccine-derived poliovirus; OPV = oral poliovirus; VDPV = vaccine-derived poliovirus; VP1 = viral protein 1.

* In the column “Outbreaks/Emergences,” outbreaks list total cases clearly associated with cVDPVs, emergences indicate independent cVDPV outbreaks, and names of emergences designate the country and geographic subnational region of the emergence and the number of emergences in each subnational region.

^†^ Total years detected for previously reported cVDPV outbreaks.

^§^ During January 2020–June 2021 with data as of November 9, 2021. For AFP cases, the number of AFP cases with a VDPV-positive specimen or in which a direct contact of the case had a VDPV-positive specimen when the case did not; for other human sources, the number of contacts or healthy children with a VDPV-positive specimen; for detections from environmental surveillance, the total VDPVs detected from environmental (sewage) collections.

^¶^ Contacts and healthy child specimen sampling during January 2020–June 2021 with data as of November 9, 2021 for all emergences.

** Percentage of divergence is estimated from the number of nucleotide differences in the VP1 region from the corresponding parental OPV strain.

^††^ For AFP cases, dates refer to date of paralysis onset; for contacts, healthy children, and environmental (sewage) samples, dates refer to date of collection during January 2020–June 2021 with data as of November 9, 2021.

^§§^ Dashes indicate data were not cumulative.

## Detection of cVDPV2

**FIGURE 2:**
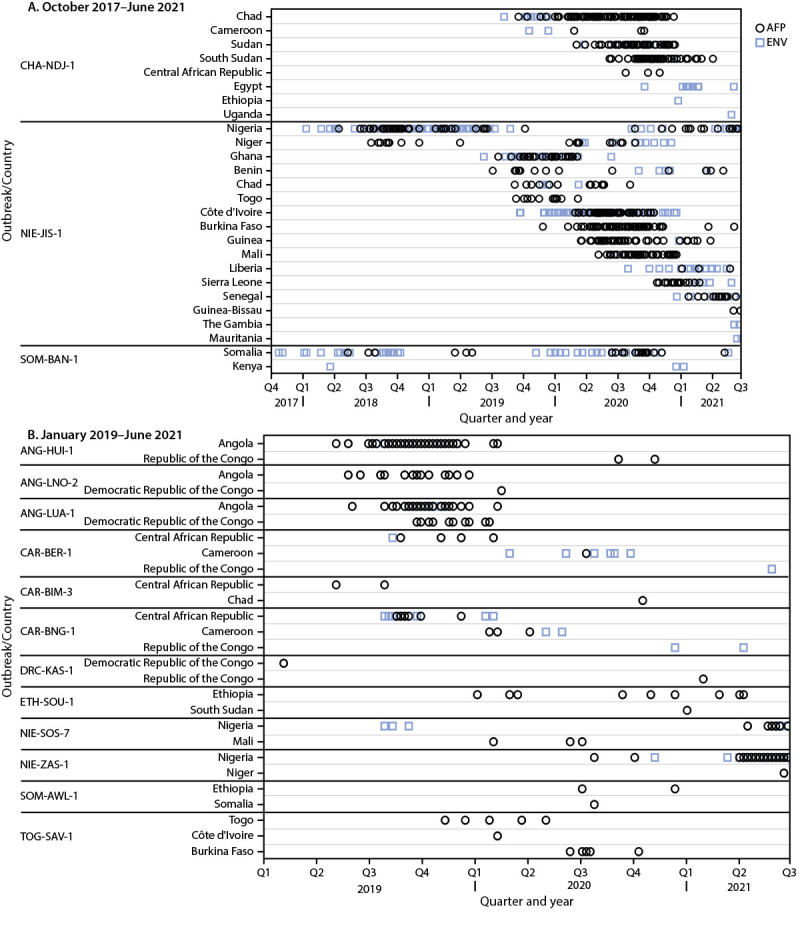
Acute flaccid paralysis cases and environmental samples positive for circulating vaccine-derived poliovirus type 2 associated with outbreaks ongoing during January 2020–June 2021 that involved international spread since emergence, by outbreak and country — Africa, October 2017–June 2021 (A)*^,†^and January 2019–June 2021 (B)*^,†^ **Abbreviations**: AFP = acute flaccid paralysis; ENV = environmental samples. * Dates (quarter and year) refer to the date of paralysis onset of AFP cases; ENV (sewage) dates refer to date of collection. When dates are the same, symbols will overlap; thus, not all isolates are visible. Outbreaks are illustrated for the country where the emergence was first detected and for countries where outbreaks with genetically linked virus were ongoing during January 2020–June 2021. † Data as of November 9, 2021.

During January 2020–June 2021, there were 38 cVDPV2 emergences in active transmission in 34 countries; 28 (82%) of these countries are in Africa (Table) (Figure 1). Nineteen (50%) of the 38 emergences were previously detected during 2017–2019, three (8%) (ETH-ORO-4, ETH-SOU-2, and NIE-SOS-7) were newly detected in 2019 but were confirmed after the last global report, and 16 (42%) were newly detected during 2020–2021 (*1*,*3*). During the reporting period, fifteen (58%) of the 26 emergences in active transmission in African countries were detected, either in AFP patients or through environmental surveillance, outside of the country of first isolation of genetically linked virus (Figure 2). No polioviruses genetically linked to two previously described emergences (CHN-XIN-1 and ZAM-LUA-1) have been detected since 2019 (*1*,*3*).

**Western Africa.** The previously described cVDPV2 emergence (NIE-JIS-1) (*1*,*3*), first detected in Nigeria in 2018, continued to circulate during the reporting period. Since first detected, genetically linked virus has circulated in 17 west and central African countries, from Mauritania to Cameroon; during the reporting period; circulation was documented in 16 of the 17 countries (excluding Cameroon) resulting in 310 cases of cVDPV2 in 14 countries and detection through environmental surveillance in 13 countries (*1*,*3*). The most recent detection of the previously described NIE-KGS-1 emergence was through environmental surveillance in January 2020 (*1*,*3*).

During July–September 2019, the NIE-SOS-7 emergence was detected through environmental surveillance in Nigeria; circulation was confirmed after the previous global update (*3*). Virus genetically linked to the NIE-SOS-7 emergence was detected in specimens from AFP patients and from one healthy child in Mali during 2020. NIE-SOS-7 was not detected in Nigeria during 2020; however, genetically linked virus was isolated in 2021 from specimens obtained from AFP patients and healthy children, and through environmental surveillance. Two new cVDPV2 emergences (NIE-SOS-8 and NIE-ZAS-1) were detected and circulated in Nigeria during the reporting period, with the most recent detections in September 2020 and June 2021, respectively. During June 2021, NIE-ZAS-1 emergence was detected in Niger. There was no evidence of continued circulation of any other previously described emergences first detected in Nigeria (*1*,*3*). The previously reported TOG-SAV-1 cVDPV2 emergence circulated in Burkina Faso, Côte d’Ivoire, and Togo during the reporting period (*3*).

**Central Africa**. The most recent detection of the ANG-HUI-1 emergence in Angola was in February 2020; however, genetically linked virus was isolated from specimens collected from AFP patients and one healthy child during late 2020 in the Republic of the Congo (*1,3*). The ANG-LUA-1 emergence was most recently detected in the Democratic Republic of the Congo and Angola in specimens from AFP patients with paralysis onset in January and February 2020, respectively and in a healthy child in the Republic of the Congo in October 2020 (*3*). The ANG-LNO-2 emergence was last detected in Angola in December 2019; the most recent isolation of genetically linked virus was in the Democratic Republic of the Congo from specimens from an AFP patient with paralysis onset in February 2020 (*1*,*3*). No polioviruses genetically linked to two previously described emergences (ANG-LNO-1 and ANG-MOX-1) were detected during the reporting period (*1*,*3*). 

The CHA-NDJ-1 emergence was first detected in Chad and then Cameroon during 2019; genetically linked virus was detected during the reporting period in Cameroon, the Central African Republic, Chad, Egypt, Ethiopia, South Sudan, Sudan, and Uganda (*3*). Genetically linked virus was most recently detected in Egypt and Uganda through environmental surveillance during June 2021. This emergence resulted in 204 paralytic cases in five of these eight countries during the reporting period.

Of the seven emergences first detected in the Central African Republic during 2019 (CAR-BAM-1, CAR-BAM-2, CAR-BER-1, CAR-BIM-1, CAR-BIM-2, CAR-BIM-3, and CAR-BNG-1), three (CAR-BER-1, CAR-BIM-3, and CAR-BNG-1) continued to circulate and spread internationally during the reporting period (*1*,*3*). Virus genetically linked to CAR-BER-1 was detected in Cameroon, the Central African Republic, and the Republic of the Congo; to CAR-BIM-3 was detected in Chad; and to CAR-BNG-1 was detected in Cameroon, the Central African Republic, the Republic of the Congo, and the Democratic Republic of the Congo.

Two previously described emergences (DRC-KAS-1 and DRC-KAS-3) detected in the Democratic Republic of the Congo in 2019 continued to circulate (*1*,*3*). After being first detected in 2019 in specimens from an AFP patient and healthy children (*1*), the DRC-KAS-1 emergence was not detected again until early 2021 in the Republic of the Congo in the specimens from an AFP patient. During the current reporting period, the DRC-KAS-3 emergence resulted in 82 paralytic cases in the Democratic Republic of the Congo, with the most recent paralysis onset in April 2021. Three new emergences (DRC-EQT-1, DRC-MAN-2, and DRC-TPA-2) were detected during the reporting period. There was no evidence of continued circulation of any other previously described emergences first detected in the Democratic Republic of the Congo (*1*,*3*).

**Horn of Africa.** The previously described SOM-BAN-1 emergence continued to circulate during the reporting period; genetically linked virus was detected each year during 2017–2021 in Somalia, and during 2018 and 2020–2021 in neighboring Kenya (*1*,*3*). During 2020, a new emergence (SOM-AWL-1) resulted in one case in Somalia and two cases in Ethiopia. Three previously described cVDPV2 emergences (ETH-ORO-1, ETH-ORO-2, and ETH-ORO-3) detected in Ethiopia in 2019 were detected during the reporting period in Ethiopia and Somalia (*3*). Two new emergences (ETH-ORO-4 and ETH-SOU-2) were confirmed after the previous global update (*3*) and subsequently resulted in six paralytic cases in Ethiopia. During 2020–2021, an additional new emergence (ETH-SOU-1) that circulated in Ethiopia and South Sudan resulted in ten paralytic cases. There have been no detections of the previously described ETH-SOM-1 emergence since 2019 (*3*).

**Afghanistan, Iran, Pakistan, and Tajikistan.** Among the five previously described cVDPV2 emergences detected in 2019 in Pakistan (PAK-GB-1, PAK-GB-2, PAK-GB-3, PAK-KOH-1, and PAK-TOR-1) only PAK-GB-1 and PAK-TOR-1 continued to be detected during the reporting period (*3*). The latest detection of PAK-TOR-1 was in a healthy child in Pakistan in early 2020. During the reporting period, PAK-GB-1 spread internationally resulting in a total of 251 cases in Afghanistan and Tajikistan, and 114 cases in Pakistan. There have been 11 environmental surveillance isolations of PAK-GB-1 in Iran, but no paralytic cases. During the reporting period, seven cVDPV2 emergences (PAK-FSD-1, PAK-FSD-2, PAK-KAM-1, PAK-KHI-2, PAK-LKW-1, PAK-PWR-1, and PAK-ZHB-1) were newly detected in Pakistan resulting in 15 paralytic cases; two cVDPV2 emergences (AFG-HLD-1 and AFG-NGR-1) were newly detected in Afghanistan during 2020 and spread to Pakistan. An additional cVDPV2 emergence (PAK-PB-1) was first and most recently detected through environmental surveillance in Pakistan in December 2019; confirmation of circulation occurred after the last global report (*3*).

**Malaysia and the Philippines.** The most recent detection of the PHL-NCR-1 cVDPV2 emergence in the Philippines was in January 2020 (*3*). The most recent detection of this emergence globally was through environmental surveillance during February 2020 in Malaysia (*3*). 

## Detection of cVDPV3

The most recent isolation of the CHN-SHA-1 cVDPV3 emergence, the only cVDPV3 in transmission during the reporting period, was through environmental surveillance in January 2021 in China (Table) (Figure 1). No paralytic cases were reported as of November 9, 2021.

## Outbreak Control

As of October 31, 2021, no transmission was detected for >12 months for outbreaks in certain countries related to three cVDPV1 and 46 cVDPV2 emergences that circulated during 2018–2020, indicating probable interruption of transmission in those countries (>12 months since the most recent date of paralysis onset in an AFP patient, or of collection of environmental surveillance sample or other sample [e.g., healthy child], positive for genetically linked virus as of October 31, 2021) (*1*,*3*,*9*) (Table) (Supplementary Table; https://stacks.cdc.gov/view/cdc/112105). In addition, as of October 31, 2021, there have been no genetically linked isolations for 7 to 12 months, indicating possible outbreak cessation of AFG-HLD-1 in Afghanistan; TOG-SAV-1 in Burkina Faso; CHA-NDJ-1 in the Central African Republic, Chad, Ethiopia, and Sudan; CAR-BIM-3 in Chad; CHN-SHA-1 in China; NIE-JIS-1 in Côte d’Ivoire, Mali, and Niger; CAR-BNG-1 in the Democratic Republic of the Congo; ETH-ORO-1, ETH-ORO-3, and SOM-AWL-1 in Ethiopia; MAD-SUO-1 in Madagascar; PAK-FSD-1, PAK-KAM-1, PAK-KHI-2, PAK-LKW-1 and PAK-ZHB-1 in Pakistan; ANG-HUI-1, ANG-LUA-1, and DRC-KAS-1 in the Republic of the Congo; ETH-SOU-1 in South Sudan; PAK-GB-1 in Iran; SOM-BAN-1 in Kenya; and YEM-SAD-1 in Yemen (*1*,*3*).

## Discussion

During January 2020–June 2021, GPEI continued to be challenged by cVDPV outbreaks, 86% of which were type 2 outbreaks affecting 28 African countries. The SOM-BAN-1, NIE-JIS-1, and CHA-NDJ-1 cVDPV2 emergences first detected in 2017, 2018, and 2019, respectively have continued to circulate well beyond the countries of first detection; these and numerous other old and new emergences have cumulatively resulted in 1,293 paralytic cVDPV2 cases during the reporting period (*1*,*3*).

Disruptions in AFP and environmental surveillance, partly because of the COVID-19 pandemic, might have resulted in case undercounts and delayed cVDPV2 outbreak detection during the reporting period (*3*,*8*,*10*). Outbreak response supplementary immunization activities were suspended during March–June 2020 (initial months of the COVID-19 pandemic) (*8*). Many outbreak response supplementary immunization activities conducted before and after the suspension have been of poor quality, and, in many countries, there have been delays of weeks to months in supplementary immunization activities implementation after outbreak confirmation, all leading to lingering and geographically expanding cVDPV2 transmission and seeding of new emergences (*1*,*3*,*8*).

A goal of the GPEI 2022–2026 Strategic Plan is to interrupt all cVDPV2 transmission by the end of 2023 by better addressing the challenges to early outbreak detection and effective outbreak responses (*8*). Initial nOPV2 outbreak response supplementary immunization activities, anticipated for late 2020 after the Emergency Use Listing was announced, were delayed until March 2021 (*3*,*6*,*8*); to date approximately 100 million nOPV2 doses have been administered in seven countries (Benin, Liberia, Niger, Nigeria, the Republic of the Congo, Sierra Leone, and Tajikistan) (*6*). The improved genetic stability of nOPV2 over that of the Sabin vaccine strain and its effectiveness in interrupting cVDPV2 transmission are being monitored because this vaccine is now authorized for wider use (*6*). In the interim, the initiative is confronted with multiple cVDPV2 outbreaks and limited nOPV2 supply because of manufacturing delays resulting from the COVID-19 pandemic and larger than anticipated nOPV2 consumption (*6*). Therefore, the recommendation from the Strategic Advisory Group of Experts on Immunization,^¶ ^WHO Director-General’s Emergency Committee for the International Health Regulations regarding the spread of poliovirus as a Public Health Emergency of International Concern (*9*), and the GPEI Independent Monitoring Board** is that countries should initiate rapid outbreak response with available type 2 OPV, whether that is Sabin or the novel vaccine (*6*).

**Summary**

**What is already known about this topic?**

Circulating vaccine-derived polioviruses (cVDPVs) can emerge in settings with low poliovirus population immunity and cause paralysis.

**What is added by this report?**

During January 2020–June 2021, 44 cVDPV outbreaks were ongoing, resulting in 1,335 paralytic cases; 38 (86%) were cVDPV type 2 (cVDPV2). Initial use of novel type 2 oral poliovirus vaccine (OPV), modified to be more genetically stable than Sabin strain poliovirus, began in March 2021 for cVDPV2 outbreak responses; current supplies are limited.

**What are the implications for public health practice?**

A goal of the Global Polio Eradication Initiative’s 2022–2026 Strategic Plan is to better address the challenges to early cVDPV2 outbreak detection and initiate prompt and high coverage outbreak responses with available type 2 OPV to interrupt transmission by the end of 2023.

## Footnotes

* In this report, a cVDPV outbreak is defined as two or more independent isolations of genetically linked VDPVs (through AFP or environmental surveillance, or from healthy community members among themselves or following confirmation of a VDPV-positive specimen from an AFP case in a person with whom they are associated). The number of outbreaks is equivalent to the number of cVDPV emergences. In summaries in this report, a given cVDPV emergence is counted once regardless of the number of countries affected after transmission beyond international borders. For the GPEI, an emergence detected in a country is considered an outbreak for that country.

† Data as of November 9, 2021 for all emergences.

§ Names designate the country and geographic subnational region of the emergence and the number of emergences in each subnational region.

¶
http://apps.who.int/iris/bitstream/handle/10665/341623/WER9622-eng-fre.pdf

**
https://polioeradication.org/wp-content/uploads/2021/07/20th-IMB-report-20210631.pdf
